# Architect Insights: Implications and Barriers of Future-Proofing Hospitals

**DOI:** 10.1177/19375867251374676

**Published:** 2025-09-16

**Authors:** Sanaz Memari, Tuba Kocaturk, Mirjana Lozanovska, Fiona Andrews, Richard Tucker

**Affiliations:** 1 Faculty of Science, Engineering and Built Environment, School of Architecture and Built Environment, 206228 Deakin University, Geelong, Victoria, Australia; 2 Faculty of Health, School of Health and Social Development, 95522 Deakin University, Burwood, Victoria, Australia

**Keywords:** hospital building design, hospital architecture, future proofing, adaptability, resilience

## Abstract

**Aim:**

This study explores how future-proofing is understood and applied in hospital building design, focusing on the perspectives of experienced architects. It aims to examine the practical implications of future-proofing and to identify key barriers to its implementation within contemporary healthcare infrastructure projects.

**Background:**

Existing literature often focuses on the general benefits, such as cost savings and sustainability, but lacks detailed analysis of the multifaceted implications and obstacles encountered in real-world projects. This study addresses this gap by directly examining architects’ perspectives, offering critical insights into the practical realities and complexities of future-proofing hospital buildings, thereby contributing to a more nuanced understanding and informed decision-making in this field.

**Methods:**

Sixteen semistructured interviews were conducted with experienced hospital architects based in Australia. Data were analyzed using a combination of deductive and inductive thematic analysis.

**Results:**

The results of thematic analysis have been categorized under three overarching categories: perceived benefits, perceived drawbacks, and implementation challenges. The findings highlight that future-proofing is neither inherently beneficial nor burdensome, but rather a context-sensitive strategy that must be tailored to each project's evolving operational, economic, and policy.

**Conclusions:**

The study underscores the need for more systematic, longitudinal evaluation of future-proofing strategies, as well as greater integration of advanced futures methodologies into healthcare planning and design processes. A more structured and evidence-based approach to future-proofing can support the development of hospital infrastructure that is both resilient and responsive to the long-term evolution of healthcare systems.

## Introduction

In the current strained healthcare economy, a shift toward more efficient use of existing resources is recommended. This has redirected attention from building new hospitals to rethinking how existing infrastructure can be adapted to meet evolving demands (Wilson & Kishk, 2012)—a challenge that future-proofing directly addresses. Future-proofing for hospitals is the strategic design of buildings to enable maximum resilience and adaptability in response to changing requirements, emerging opportunities and risks, and potential shocks, with minimal disruptions. From this perspective, future-proofing not only sustains the initial value of hospital buildings but also creates added value, creating buildings with multiple life cycles. In addition, future-proofing integrates both technical and social considerations, positioning hospital buildings not only as passive shields against disruptions but also as active agents that can positively shape healthcare delivery and well-being (Memari et al., 2023).

Hospitals are particularly vulnerable to rapid physical deterioration due to their continuous operation and complex, evolving functional demands (Jandali et al., 2018; Olanrewaju et al., 2018). These conditions underscore the need for robust future-proofing strategies capable of navigating a multifaceted and constantly shifting landscape of change. Such change drivers can be broadly categorized as industry-specific (e.g., healthcare system transformation including shifts from inpatient to outpatient care and the rise of telehealth; changes in medical technology such as advancements in diagnostic imaging and robotic surgery; public health crises necessitating adaptation for pandemics; and evolving infection control standards, etc.), context-specific factors (e.g., the influence of associated systems regarding location such as local infrastructure and zoning regulations; organizational culture influencing facility design; demographics such as an aging population; environmental considerations including climate change impacts and energy efficiency; and local economic conditions) and time-specific factors (e.g., associated with the life cycle performance deterioration of structural and mechanical systems due to continuous operation; regulatory updates over time requiring retrofits; and accumulated maintenance debt leading to large-scale renovation needs) (Memari et al., 2022).

The dynamic interplay of internal and external factors in hospitals compels us to recognize them as critical infrastructure (Kendall, 2018). Like other infrastructure, hospitals serve multiple users, operate continuously, and require ongoing maintenance or upgrades while maintaining overall functionality. Scholars have called for a comprehensive approach to assessing hospital building lifespan quality. This assessment should prioritize factors such as low operational costs, adaptability, and durable materials, moving beyond the current emphasis on completion time and capital cost (Bjørberg & Verweij, 2009; Nedin, 2013). Such an approach evaluates how a hospital building sustains healthcare services over time and generates life cycle value. Recognizing this capacity for long-term service delivery reframes hospitals not just as fixed facilities but as dynamic assets integral to healthcare performance. Therefore, hospitals should also be viewed as valuable assets that significantly influence healthcare services and outcomes; they possess the agency to act as pivotal elements in delivering healthcare services by shaping patient experiences, optimizing space for advanced medical technologies, and offering adaptable environments that can be reconfigured to meet future healthcare demands, thereby aligning the physical structure of healthcare facilities with strategic organizational and operational goals.

Thoughtful design, construction, management, and maintenance are essential to ensure hospital buildings are future-proof and remain fit for purpose (Clark, 2015). Therefore, future-proofing is often perceived as a key quality contributing to the overall success of hospitals (Brambilla et al., 2020) and a viable solution to mitigate obsolescence. Nonetheless, future-proofing hospital buildings require a delicate balance. It must be “both broad enough to include variations and narrow enough to be justifiable from a project cost and delivery perspective” (Karlsson et al., 2021, p. 225). Due to its complexity, future-proofing practice presents challenges in justifying future benefits (Götze et al., 2015), ensuring value at every phase of life cycle (Love et al., 2015), and making business-case due to uncertainty and complexity associated with the future (Kafuku et al., 2015).

While previous research has predominantly emphasized capital investment as the primary barrier to future-proofing, this study adopts a critical lens, delving into the multifaceted implications of this practice through in-depth expert interviews with hospital architects, aiming to provide a comprehensive understanding of the implications of future-proofing in hospital building design and to identify the common obstacles encountered when implementing such an approach.

### What Is Future-Proofing?

In general, future-proofing is a life cycle and process-oriented perspective addressing emerging dynamic requirements (Love et al., 2015) that can help prevent capability gaps—deficiencies or mismatches in an asset's ability to meet evolving needs—that develop over time (Rehman & Ryan, 2018). As such, Krystallis (2016) defines future-proofing as a dynamic system approach that can address not only negative situations but also accommodate positive opportunities. This perspective redefines change, often seen as a negative force, as an opportunity to be embraced. In this light, future-proofing represents a shift from cost-minimizing design practices, focused primarily on reducing initial construction and maintenance costs, to innovative approaches aimed at “maximising whole-life value in the face of unpredictable, ongoing change” (Salvado et al., 2020, p. 2) and therefore contributing to sustainability, as noted by Verhaeghe et al. (2022, p. 11): “future-proofing is an essential cornerstone in the transition towards a more sustainable built environment.”

Future-proofing is a response to uncertain future conditions, including but not limited to disruptive technological innovations, political changes with influence on healthcare budget and model of care, pandemics, and environmental disasters accelerated by climate change, whereby a building is designed to respond to future changes in requirements, uses, strategic perspectives, business drivers, policies, and climate (Krystallis et al., 2019). Therefore, it involves stress-testing building solutions against a range of potential future scenarios to ensure their functionality throughout the building's life cycle to prevent disruptive and costly refurbishments or premature decommissioning (Georgiadou et al., 2012).

On the other hand, future-proofing bridges the gap between building science and social science, as noted by Leaman et al. (2010). Memari et al. (2023) further expand this understanding by investigating future-proofing at the nexus of architectural design and foresight and future studies, where designing hospital buildings is also understood as shaping the future of healthcare delivery. Accordingly, their proposed future-proofing conceptualization, besides a *passive* role in response to threats, disruptions, imposed changes and acting as a shield, hospital buildings can act as an intervention and play an *active* role as change agents with transformative power in multiple aspects such as being a good neighbor by doing no harm, serving community providing services covering all determinants of health and well-being, and providing a health-promoting environment for patients and staff (Memari et al., 2023).

The following strategies are among the most commonly identified by architects in hospital building design (Memari et al., 2023). *Soft spaces* are areas intentionally designed to be easily converted into core hospital functions, supporting flexible use as needs evolve. *Open-ended corridors* enable future expansion by allowing extensions without significant structural alterations. The *empty chair* concept reserves portions of the site for future development, ensuring that physical space is available for anticipated growth. *Interstitial floors*—service zones inserted between main floors—offer additional capacity for mechanical systems or equipment upgrades, facilitating adaptation over time. These strategies can also be understood in relation to their temporal orientation: standardization often supports short-term flexibility; *soft spaces* enable medium-term functional shifts; and *planning for extension*, such as open-ended corridors and reserved zones, supports long-term scalability (Kyrö et al., 2019).

There is a lack of a cohesive approach to evaluate future-proofing potentials in built environments (Rich, 2016). This gap, particularly in the context of hospital buildings, hampers an informed application of future-proofing strategies (Masood et al., 2015). As such, this study conducted interviews with Australian-based hospital architects to critically examine the concept of future-proofing and to explore the practical implications and challenges of applying these strategies in hospital design. The focus is specifically on Australian public hospitals, given their essential role in delivering equitable, universally accessible, and often highly complex care to the broader population.

## Method

### Setting

This study used semistructured interviews with experienced hospital architects to investigate the challenges and implications of implementing future-proofing strategies in hospital design. The research received approval from Deakin University's Human Research Ethics Committee. Each interview lasted approximately 50 minutes and was conducted online via Zoom by the first author.

### Participants

The studies conducted by Bowen (2008) and Guest et al. (2006) indicate that saturation typically occurs after about 12 interviews. To ensure this, sixteen semistructured interviews were conducted in this study. Purposive sampling from the authors’ professional networks and progressive snowball sampling (referrals from interviewees) were used (Groenland & Dana, 2019). The final sample comprised 16 architectural practitioners from seven leading Australian firms, located across Western Australia, Queensland, New South Wales, and Victoria. Participants were selected based on the following four criteria:

- Australia-based practitioners—to ensure relevance to the Australian healthcare context.
- Architects involved in healthcare design—since their role includes integrating stakeholder inputs into hospital projects.
- Professionally experienced in healthcare buildings—with over fifteen years of hospital design experience.
- Familiarity with future-proofing—highlighted in their professional biographies or firm visions, reflecting their engagement with future-proofing strategies and challenges.

Based on these criteria and referrals from the first five interviewees, 24 hospital architects were identified and invited to participate. Each received an email invitation that included a Plain Language Statement and Consent Form. Of these, 16 agreed to take part in the study.

### Interview Guide

The interview guide was structured around three primary questions, each accompanied by targeted prompts:

1. How critical is a future-proofing approach in hospital architecture?
  *Prompt:* Why? What are the consequences of not future-proofing hospital buildings?
2. What are the advantages and disadvantages of embedding a future-proofing approach in the architectural design process of hospital buildings?
  *Prompt:* Could you please give any successful and unsuccessful examples from hospital projects?
3. What have been the difficulties in embedding a future-proofing approach in the architectural design process of hospital buildings?
  *Prompt:* What would be possible solutions to address these difficulties?

Each interview lasted approximately 50 min, was conducted online, recorded, and transcribed by the first author.

### Analysis

Data were analyzed using content analysis (Vaismoradi et al., 2016). Initially, a deductive approach was used to code the interview data into three main categories: Drawbacks, Benefits, and Challenges. Following this, an inductive approach was employed, with the first author initially conducting thematic analysis to identify and explore key patterns and themes within each category, which were then discussed and validated by the second, third, and fifth authors. After initial data analysis, the member-checking technique (Koelsch, 2013) was employed to ensure the validity and applicability of the research findings. Member checking, also known as respondent validation (Birt et al., 2016), involves presenting the analyzed data back to the participants for their feedback. As a result, the initial interview analysis was confirmed by the interviewees with minor recommendations for further clarity regarding the terminology.

## Findings

The findings are organized into three main categories reflecting the perspectives of interview participants: the drawbacks, benefits, and challenges of applying future-proofing strategies in hospital design. Each of the following subsections elaborates on these categories in greater detail.

### Drawbacks of Future-Proofing Hospital Buildings

Interviewees frequently described future-proofing as a resource-intensive endeavor, raising concerns about the significant upfront investments required. Architect #5 highlighted the weight of capital costs, while Architect #16 described these costs as a “penalty,” reflecting the perceived financial burden placed on projects. Beyond capital expenditure, Architect #10 emphasized the considerable time, energy, consultation, and research needed to effectively implement future-proofing strategies. Despite these concerns, several architects, including Architects #5 and #16, stressed the importance of viewing these investments through the lens of long-term return on investment (ROI). Architect #4 acknowledged the reality of “extra expenditure” that may not yield immediate or short-term benefits, underscoring the need to balance capital costs against anticipated long-term gains. These reflections highlight the importance of careful evaluation. Future-proofing decisions must be guided by comprehensive analyses that weigh upfront costs against the potential to generate lasting value—both in financial and operational terms—over the building's life cycle.

Interviewees also discussed that these dynamic challenges may potentially broaden the conventional scope of architects’ work, necessitating the acquisition of new skills and expertise, using methodologies used in futures studies such as scenario planning and backcasting. The interviewees also highlighted that increased space allocation due to future-proofing can lead to unfavorable consequences like longer corridor distances from bedrooms affecting efficient serviceability (Architect #1) and increased operational expenses for cleaning and maintenance of larger facilities (Architect #6). Architect #16 echoed these concerns, noting significant financial burdens on the operational cost of big hospitals. Prioritizing versatility and adaptability in future-proof designs might sacrifice some efficiency compared to designs tailored to current, specific needs. This approach may require allocating extra resources or space beyond immediate requirements to prepare for potential future needs (Architect #16).

Beyond operational costs, the findings revealed that redundancy plays a significant role in future-proofing and can substantially influence maintenance expenses. For example, Architect #1 emphasized the importance of anticipating specific risks and vulnerabilities through built-in redundancies—elements that often incur considerable maintenance costs, especially when they are not regularly utilized (Architect #1).

### Benefits of Future-Proofing Hospital Buildings

Infuture-proofing, buildings are viewed as strategic investments. The interviewees underscored the importance of future-proofing hospital facilities to safeguard the substantial investments made in their construction, maintenance, and ongoing development.

Architect #16: A lot of money has been invested in building them [hospital buildings], a lot of money has been invested in maintaining them, a lot of money has been made to put into changing and evolving them over time. So future-proofing by prolonging the functional life of buildings saves all the investments.

From a financial point of view, future-proofing could minimize the cost of reconfiguration across the whole life cycle: “Do you have to redo everything, or can you accommodate that change in a cost-efficient” (Architect #6) and “it [future-proofing] reduces the cost of the future building changes” (Architect #9). For instance, “it [future-proofing] could have been done for $3 million, but later on it might cost you $20 million to rectify. So, there's an opportunity cost with it” (Architect #12). Furthermore, as noted by Architect #12, implementing future-proofing measures can minimize disruptions and downtimes, allowing for a rapid response to necessary changes. Overall, the interviewees suggested that the expense of implementing future-proofing is negligible in comparison to the consequences of not implementing it:

Architect #16: So you're actually paying a little bit of a premium in terms of building costs to avoid disruption down the track … sometimes they don't cost more really. Obviously, things like vertical expansion might cost a little bit more, but it's a really minor percentage on what the overall cost of the building is.

Architect #12: It's a very minor cost. We calculated on the last project that we did a huge amount of future-proofing, and it was a cost of around 3%.

In addition to economic benefits, it was suggested that future-proofing enhances environmental sustainability by extending the useful life cycle of facilities, which reduces waste from demolition and reconstruction and minimizes operational disruptions caused by redevelopment. Architect #4 noted that effectively implementing future-proofing at different scales can delay the need for additional construction in future projects that may not be immediately necessary.

Architect #4: If it [future-proofing] is done well at different scales, it means you can defer the next [redevelopment] project. You are enabling future projects deferring capital towards being available.

As Architects #2 and #4 noted, future-proofing offers advantages not only in addressing long-term life cycle requirements but also during the design and construction phases by integrating flexibility to maintain open possibilities, even in the event of design modifications necessitated by changes in project briefs or adjustments during construction. Additionally, Architect #16 observed that future-proofing can deliver significant benefits during a building's initial operational phase by accommodating emerging clinical needs and spatial reconfigurations. This flexibility is especially valuable given the long timelines involved in healthcare design and construction, helping to ensure the facility remains adaptable and aligned with evolving requirements.

### Challenges in Future-Proofing Practice

In addition to understanding the benefits and drawbacks of future-proofing, it is crucial to examine the barriers that hinder its practical implementation in hospital building design. This section presents five interrelated categories of challenges identified through the interviews: the complexity of hospital architecture, misconceptions about future-proofing, limitations in planning methodologies, gaps in data accessibility, and financial and institutional constraints.

#### Navigating Complexity in Hospital Architecture

The findings reveal that one of the primary challenges in future-proofing hospital buildings lies in the inherent complexity of the typology itself. Hospitals function as intricate systems characterized by competing priorities, numerous interdependencies, and constrained resources. These factors often make it difficult for design teams to look beyond immediate demands and creatively anticipate the future needs of healthcare environments:

Architect #3: In a lot of cases, we don't get imaginative enough about where hospital infrastructure needs to head. We’re too rooted in today's problems, the budgets, programs and what we can do now … There are so many issues to juggle.

This complexity is compounded by the high degree of interconnection within hospital systems. Interviewees used metaphors such as “the ripple effect of change” (Architect #12) and a “Rubik's cube” (Architect #2) to illustrate how seemingly isolated changes can have wide-ranging implications. For instance, Architect #9 explained that adding a new wing—such as additional wards—can affect other areas like public toilets and kitchens, often necessitating broader capacity upgrades rather than simple expansions. This interconnectedness also complicates long-term planning. The constant need to respond rapidly to immediate crises can divert attention and resources away from enduring challenges such as climate change. As Architect #1 noted, the urgency of the COVID-19 pandemic overshadowed critical conversations around long-term environmental planning, highlighting the importance of maintaining focus on both immediate and future priorities.

Operating 24/7, hospitals must also contend with the difficulty of implementing changes without disrupting essential services. As Architect #7 observed, minimizing downtime is critical to ensuring continuous healthcare delivery. Architect #16 added that construction-related impacts—such as noise, dust, and disruptions—can significantly affect patient outcomes, underscoring the need for careful staging and decanting strategies when redeveloping existing facilities.

Finally, interviewees pointed to a persistent trade-off between long-term flexibility and short-term cost-efficiency. Architect #4 noted that large, consolidated hospital buildings may be cost-effective in the short term but become difficult to adapt over time:

Architect #4: We do tend to produce buildings that are very aggregated in big lumps and that leads to challenges later. They're more cost-efficient and effective … But when they're very interconnected, it's hard to then take apart and refit one area at a time or change it … compared with a number of buildings [that] are there more loosely connected [enabling future-proofing].

The findings also highlight a key challenge in future-proofing hospitals: many projects are brownfield developments rather than greenfield sites, meaning design strategies are often constrained by the limitations of existing structures. For instance, upgrading facilities in these older buildings can be nearly impossible (Architect #6); the reason is “what they've done in the past” (Architect #9), which ultimately led to “demolishing buildings that are three years old because they are in the wrong spot” (Architect #12). In addition, deep plan, bespoke design (Architect #16), precast concrete, and nonstandardized grids (Architect #3) were recognized as obstacles limiting future changes and redevelopments in their projects.

#### Misconceptions and Misapplications of Future-Proofing

A common perception that future-proofing is inherently capital-intensive often leads to its deprioritization during business case development and cost–benefit analysis. Several architects noted that maintaining design flexibility within budget constraints is a persistent challenge. Architect #5 observed that future-proofing considerations are frequently excluded early in the planning process due to the widespread belief that flexibility inevitably comes at a high cost. This misconception discourages investment, particularly when competing priorities dominate, resulting in future-proofing strategies being overlooked or undervalued. This aversion has historical roots. Architect #7 recalled that during the 1970s, large-scale shell space expansions were common but ultimately became associated with inefficiency and unjustifiable costs. As these projects proved difficult to defend economically, the idea of investing heavily in flexibility without a clear, immediate benefit gained a negative reputation—further complicating the integration of future-proofing into contemporary hospital design. To counter these misconceptions, several interviewees clarified what future-proofing is—and is not. Architect #4 explained that genuine future-proofing is not about designing the most expansive or technologically advanced building possible. Rather, it involves crafting a strategic framework that allows for localized, incremental change over time. Avoiding irreversible decisions and enabling adaptability without overspending were seen as key principles. Architect #7 similarly emphasized that future-proofing is about enabling evolution, not overproviding for every possible scenario. It requires targeted, strategic design choices that accommodate future uncertainty without burdening projects with excessive upfront investment.

The changing nature of healthcare delivery further challenges traditional assumptions. Architect #12 noted that in the past, large hospitals were viewed as the default response to rising demand. However, Architect #7 pointed out a significant shift over the last 25 years: hospitals now operate with fewer beds while managing higher patient throughput, with over 50% of patients discharged the same day—compared to just 10–20% in earlier decades. These shifts invite a reassessment of what future-proofing should look like today. As Architect #10 put it:

“If we look at now, compared with 30 or 40 years ago, you might find that we don't need the large central tertiary hospital complexes … because the predictions are that you'll be able to do a lot more facilities and treatment in the home or specialist centres.”

Likewise, Architect #4 suggested that “the trend now is not to make things bigger and bigger and more endlessly flexible, but to make them appropriate,” while Architect #16 concluded: “Making it [a hospital] big isn't always good—making it the right size may be perfect.” This points to a broader reconsideration, as Architect #4 observed:

“There's a reconsideration going on there about what is big enough, good enough for general use in the future.”

#### Limitations in Planning Methodologies for Uncertainty

Most interviewees identified uncertainty about the future as a central challenge in designing future-proof hospital buildings. Architect #6 acknowledged the inherent difficulty of accurately anticipating future needs, noting that long-term predictions are often proven wrong. Architect #16 echoed this sentiment, observing that historical comparisons between hospital redevelopments and their original master plans frequently reveal substantial divergence. This unpredictability led interviewees to characterize future-proofing as “a bit of a crystal ball” (Architect #3), “guesswork” (Architect #15), and “anecdotal” (Architect #1). As Architect #3 explained: “There's a lot of grey area in determining what is an appropriate level of future-proofing. It's a bit of a crystal ball. So I think the challenge to the design team is, what level of future-proofing can we put in place?” Even when robust evidence exists, interviewees noted that certain less frequent or politically contentious future scenarios may be disregarded. For instance, Architect #1 remarked that climate change planning is sometimes dismissed because “some people still don't believe in climate change.”

Several architects also highlighted the risks associated with relying on inaccurate future predictions, which can misguide design assumptions and lead to poor outcomes. Architect #1 cautioned that overemphasizing resilience based on flawed assumptions can undermine other critical functionalities, resulting in designs that provide minimal tangible benefits.

The concept of future-proofing was also critiqued for its lack of methodological clarity. Architect #3 described it as a “buzzword” that lacks a systematic design approach, while Architect #1 felt it is sometimes used superficially for political correctness. Political and institutional barriers to effective future-proofing were a recurring theme. Architect #4 described how short-term responses driven by political agendas can inhibit long-term thinking. Architect #11 pointed to the pressure for rapid returns on investment, while Architect #3 noted that the demand to deliver projects quickly often compromises long-term adaptability. Conversely, Architect #1 also emphasized that future-proofing carries significant political implications, requiring difficult decisions about how to allocate resources fairly across regions. This includes balancing community expectations and identifying which populations should benefit most from resilient healthcare infrastructure.

A major structural challenge to future-proofing in Australia, according to Architect #5, is the continued reliance on outdated brief development processes derived from 1950s UK health planning models. These models, embedded in the Australasian Health Facility Guidelines, follow a linear algorithm—starting with a service model and translating it into fixed room counts and sizes. As a result, hospitals are often designed around inflexible lists rather than evolving contextual needs. This rigidity is further reinforced by top-down briefing approaches. As Architect #7 explained: “From the health department, the immediacy of solving a particular problem—X beds for X dollars.” Similarly, Architect #12 argued that “the main reason I found premature obsolescence is that future-proofing wasn't taken into account; it was designed on a very specific brief, with no provision for the future.” According to Architect #3, guidelines that emphasize specific room sizes can inhibit the adoption of modular or flexible design solutions.

Several interviewees advocated for a shift toward more responsive, bottom-up planning models. Architect #4 stressed the importance of understanding shifts in healthcare delivery models and patient needs before committing to built form, recommending space allocations that allow for future adaptation. Architect #12 added that many services currently delivered in hospitals could eventually move to alternative, nonhospital settings. Building on this, Architect #4 argued for bottom-up approaches in spatial planning that allow for greater long-term flexibility:

Architect #4: Now, demographics are understood; that data is available easily, the way in which the major providers respond to it. The most difficult one to access … clinical plans, and care plans on the community level, and the regional level … be[ing] aware of where their own gaps are; like in client flow mapping… There are trends in what is treated in a hospital, what is treated in the community, and they are usually recognized and discussed, and then the way in which care is delivered is usually recognized, recorded [and] discussed. But there's limited information about spatial allowance…We're not collecting all that data yet to map complex visitations and treatments. If we start looking at that, instead of looking at what the facility does, it's a whole another area of research to do, but it could connect to what might be a facility-based approach here with flexibility; the two will come together but it's a massive undertaking. It's something we're going to have to do.

#### Data Gaps and the Loss of Future-Proofing Knowledge

The interviewees pointed out several challenges related to obtaining and transferring future-proofing data across projects. Architect #1 noted the issue of neglecting postoccupancy evaluations, which could provide valuable insights for applying successful future-proofing strategies to future projects. On the other hand, Architects #11 and #12 discussed how conservative approaches to protecting intellectual property limit the sharing of data on successful future-proofing strategies. Moreover, Architect #7 highlighted problems with transferring data to the operational phase, resulting in the loss of information about future-proofing potential embedded in the design:

Architect #7: Communicating your thoughts to the future, to the people who will be looking after the building … [is] quite important, so that they understand that the structure has been designed for future-proofing … It's quite amazing how quickly those people change and some of that intelligence is lost. Currently, we are not good at communicating those lifecycle data.

#### Financial Constraints and Value Management Pressures

All interviewees identified value management and business case development as major obstacles to implementing effective future-proofing strategies. Architect #1 noted that future-proofing is often scaled back or discarded entirely due to its perceived high cost. Similarly, Architect #5 highlighted that financial pressures and competing priorities frequently divert attention from long-term adaptability goals. Even when future-proofing intentions are embedded in the early design stages, they are often lost or overlooked during later phases of project delivery. As Architect #4 observed, original design allowances for adaptability are frequently not implemented due to shifting priorities or budgetary constraints.

Interviewees emphasized that future-proofing is particularly vulnerable to value management processes, where decisions are often guided by immediate cost savings rather than long-term ROI. Several architects noted that the long-term benefits of future-proofing are difficult to quantify, making them appear intangible or unconvincing in cost–benefit analyses. For example, Architect #6 explained that the value of future-proofing is not always well understood by the public or decision-makers and is hard to justify when additional costs do not yield immediate, visible benefits. Architect #9 added that because future-proofing is not directly tied to clinical outcomes, it is often deprioritized. Architect #6 further stressed the challenge of demonstrating future value for money, while Architect #1 pointed to the broader issue of having to make difficult decisions about what level of future-proofing is “appropriate” in light of limited resources and competing demands. Architect #1 concluded that: “we just didn't even bother doing non-essential versus essential. We made it all essential.” As a result, high levels of redundancy, overprovision or over-engineering can make future-proofing practice cost-intensive. However, future-proofing design objectives need to align with levels of future service, as noted by:

Architect #9: The health service providers have to keep a lid on, what does my community do now? What do my staffing have the capacity to provide? … So, the healthcare service providers need to make sure that they understand what level of service they are providing and just stick to that.

Architect #7: It's sort of buried in the middle of the feasibility study area. They don't actually make it as prominent as it should be.

To address financial challenges, some hospitals have started selling off vacant land on their premises, potentially hindering future redevelopment opportunities:

Architect #3: I think that the trend over recent years has been to restrict hospital sites to as small as necessary, sell off land in cities and all this kind of stuff to make more money, and that's really short-sighted.

Table 1 provides a summary of the drawbacks, key benefits, and prevalent challenges of future-proofing in hospital buildings, identified by the interviewed architects, followed by the next section, which discusses the main findings.

**Table 1.** Summary of the Key Findings.

Category	Key insights
Drawbacks	Resource-intensive practice requiring further capital investment and design time. Costs may not be directly related to immediate and short-term benefits. Expanded scope of work for architects that necessitate acquiring new skills and expertise (e.g., futures methodologies such as scenario planning, back-casting). Unfavorable consequences from increased space allocation, such as increased operational and maintenance expenses and redundancy. Prioritizing versatility and adaptability might lead to less efficient designs compared to those tailored for immediate, specific needs.
Benefits	Minimizes reconfiguration costs over life cycle. Accommodates design modifications necessitated by changes in project briefs or adjustments during construction. Provides flexibility and agility in response to necessary changes at any stages of the operation due to emerging clinical requirements, spatial reorganization, etc. Extends the useful life cycle of facilities. Retains embodied carbon and reduces waste from demolition and reconstruction. Minimizes operational disruptions and downtimes caused by redevelopment. Defers the need for construction that may not be immediately necessary.
Challenges	*Complexity:* Hospitals’ intricate interdependencies, constant and critical operations complicate long-term vision for future planning. *Misconceptions:* Future-proofing is misunderstood as costly overprovision and precise prediction, rather than “strategic moderated resilience and adaptability.” *Underdeveloped Methods and Practice:* Outdated brief processes, top-down approaches, and a lack of application of advanced futures methodologies in planning and design process, hinder effective implementation. *Data Inaccessibility:* Poor postoccupancy evaluations and concerns about intellectual property about successful design strategies limit knowledge transfer. *Business Case Difficulty:* Lack of certainty and proof about long-term benefits make justification against immediate costs challenging, leading to deprioritization.

## Discussion

This study explored the implications—both benefits and drawbacks—of future-proofing hospital buildings, as well as the challenges associated with its implementation, based on insights from Australian hospital architects. While participants acknowledged several drawbacks of future-proofing, including higher capital costs, longer planning and design timelines, increased operational and maintenance demands, and potentially less efficient layouts compared to purpose-built designs for immediate needs, these concerns were generally outweighed by the perceived long-term value. Despite the increased upfront investment, interviewees emphasized that the additional cost of future-proofing is relatively modest when considered against the potential life cycle benefits and enduring adaptability it provides. These drawbacks are consistent with those identified in prior research, including larger room sizes, technical spaces, additional capacities (Kyrö et al., 2019), associated issues with overdesign, such as capital cost (Ross & Hastings, 2005), operational costs, and energy consumption implications (Djunaedy et al., 2011; Jones et al., 2020; Peeters et al., 2008). Oversizing in general can be criticized as it may lead to higher energy and material consumption (Verhaeghe et al., 2022). Yet, the combination of advanced computational tools and human design expertise can greatly improve the efficiency and effectiveness of hospital spatial layouts (Bayraktar Sari & Jabi, 2024). Additionally, special attention must be given to rebound effects, as for example, there is a risk that focusing extensively on enhancing a building's robustness for various extreme operational conditions could result in unnecessarily high operational costs (Li & Wang, 2022).

However, consensus is yet to be reached on the specific design requirements necessary to qualify as future-proof, robust, or similar terms (Verhaeghe et al., 2022). While drawing on best practices from successful past projects remains valuable, incorporating futures methodologies and identifying emerging signals and trends can offer deeper insights into the evolving landscape of healthcare. This approach encourages the development of foresight—a nuanced understanding of multiple possible futures—rather than relying on singular, linear predictions. In this context, hospital-centric care is increasingly viewed as unsustainable. Future hospitals are expected to evolve into specialized acute-care centers, as many healthcare functions continue to decentralize and shift into community-based settings (Rosen et al., 2010). This shift necessitates a more systematized approach to care delivery, in which hospitals function as one component within a broader, integrated patient care pathway. Their role transitions from that of a self-contained provider to a contributor responsible for just one segment of the continuum, thereby adding complexity to planning and design efforts (World Health Organization, 2018). Ultimately, future hospitals will serve as multidisciplinary “places of last resort” for complex and sophisticated interventions, requiring specialist skills and equipment (Kagioglou et al., 2010). Therefore, project briefs should adopt a “process,” “organization,” and “operational” level understanding of building usage as suggested by Macchi (2017).

The findings also highlighted several key benefits of future-proofing, including long-term economic value, cost savings on future redevelopments, uninterrupted healthcare operations, improved quality of care delivery, and enhanced environmental sustainability. By enabling adaptability and resilience, future-proofing supports long-term ROI, positioning such buildings as strategic assets rather than fixed-cost liabilities. For example, Sharma et al. (2024) demonstrate the financial logic of future-proofing through shell space planning. Cold shell spaces, adjusted by a 0.5 factor, and warm shell spaces, adjusted by a 0.35 factor of the Project BGSF (Building Gross Square Footage), illustrate the proportional cost efficiencies associated with reserving space for future use. A notable example is the Royal Children's Hospital Melbourne, where a cold shell on level five was successfully converted into a 30-bed inpatient ward in 2022—demonstrating the long-term value and flexibility of such strategies. Moreover, future-proofing can help defer unnecessary future construction, allowing healthcare organizations to focus resources on immediate priorities while retaining the capacity for strategic expansion. This approach supports both operational efficiency and financial prudence in an environment of evolving service demands.

According to the interviewed architects, future-proofing hospital design presents a range of challenges, including limited understanding and common misconceptions, inherent risks and uncertainties, financial constraints, documentation issues, constraints posed by existing structures in brownfield redevelopments, business case and value management pressures, and political influences on budget priorities. These findings align with the challenges identified by Groeneveld et al. (2018) in healthcare design projects, including practical challenges (fieldwork, involving end users, and managing sensitive situations), managerial challenges (relationship management, building understanding, and communicating value), and generic challenges (time and financial constraints, and establishing rapport). Despite the clear importance of having a whole-of-lifecycle approach to future-proofing hospitals (Carthey et al., 2011), life cycle planning can lose focus as projects progress (Schatvet et al., 2017). The findings of this research confirm inadequate focus on comprehensive life cycle planning (Bjørberg & Verweij, 2009) and poor implementation of it (Schatvet et al., 2017). With the current dominance of daily healthcare services (Støre-Valen et al., 2014), current approaches to life cycle thinking in hospital design are often limited and tend to focus only on elective phases (Thiébat, 2019), dividing the building process into three main phases—“ideation,” “construction,” and “service life.” Instead, future-proofing should integrate life cycle thinking throughout all phases—namely, raw material acquisition, product manufacturing, construction processes, building operation and maintenance, refurbishment, end-of-life deconstruction or demolition, waste processing for reuse and recycling, energy recovery, and disposal—as emphasized by Castro et al. (2015) in the context of sustainable development. In addition to evaluating time, cost, and quality immediately after completion, hospital projects should be assessed against life cycle qualities such as adaptability, the durability of building materials, and the building's capacity to accommodate evolving healthcare services over time (Bjørberg & Verweij, 2009). Memari et al. (2023) further broadened the scope of future-proofing to include both technical and social considerations across the life cycle, such as climate resilience, maintainability, circular economy, and user-centered design.

This research also highlighted how misconceptions surrounding future-proofing impede the development of a compelling business case for its adoption. These fallacies depict future-proofing as an accurate prediction (instead of foresight) and inefficient and cost-intensive practice, often influenced by past unsuccessful attempts at implementation (Pinder et al., 2013). Although our research aligns with previous findings that future-proofing can increase capital costs (by 6–12%; World Health Organization, 2009), it is often cost-effective in the long run due to reduced life cycle costs (Rehman & Ryan, 2016). This is especially significant given that operational costs typically surpass initial costs within two to three years after completion (Støre-Valen et al., 2014). Despite this, economic pressures often drive discussions toward the cheapest and quickest solutions, minimizing capital costs (Chew et al., 2004; Hareide et al., 2016; Krystallis et al., 2022). While focusing on immediate needs is understandable, short-term thinking can hinder future development (Hareide et al., 2016). Although capital investments need to be justified by future benefits (Götze et al., 2015), investment analysis can be difficult due to uncertainty and complexity (Kafuku et al., 2015). This often results in future-proofing being treated as an additional requirement rather than an integral part of the design (Masood et al., 2016). Additionally, there is a notable disconnect between understanding what needs to be future-proof and how to achieve it (Love et al., 2018). Habraken (2017) points out that the design professions lack a comprehensive body of knowledge about how built environments evolve over time. Value creation in design depends on both existing and emerging needs (Haddadi et al., 2016) and is tied to usability and adaptability (Schatvet et al., 2017). Challenges arise in defining added value merely through mathematical approaches (Hareide et al., 2016) and in finding quantitative evidence to support the benefits of future-proofing (Pinder et al., 2013). To address these challenges, Krystallis et al. (2022) suggest using Real Options Reasoning to operationalize future-proofing in complex infrastructures. Real Options Reasoning focuses on verbal theorizing rather than strict analytical modeling to develop and test hypotheses (Trigeorgis & Reuer, 2017), offering a more nuanced understanding of future-proofing in infrastructure development (Krystallis et al., 2022). Therefore, it is recommended that effective future-proofing should incorporate various futures thinking research methods as a core element of the design process (Evans & Sommerville, 2007). Design methodologies should be constantly updated using advanced future methodologies, which not only enhance future thinking but also improve innovation and design thinking.

The goal of future-proofing is not to predict the future with precision but to prepare for a broad range of possible futures. A common misunderstanding arises when future-proofing is conflated with forecasting, often driven by the desire for certainty in a highly regulated, capital-intensive healthcare environment. This pressure can lead to overly linear thinking, where past trends are simply projected forward. In contrast, effective future-proofing requires an acknowledgment of the dynamic and evolving nature of healthcare systems, technologies, and societal needs. As noted by Memari (2022, p. 134) “limited futures literacy and limited usage of futures thinking research methods during the planning and design phases of hospital buildings have resulted in the perception of future-proofing as anecdotal and guesswork.” While future-oriented thinking is often dismissed as prescience, implying that it is nonscientific in nature (Gümüsay & Reinecke, 2024), by studying available frameworks, tools, and techniques for futures thinking, researchers can equip design teams with the knowledge to anticipate and respond to dynamic and uncertain future needs. This emphasis will ensure architectural practice is not only reactive but also proactive in fostering sustainable and resilient communities.

Short-term thinking and politically driven emphasis on inpatient bed numbers—often in response to immediate pressures—were identified as significant barriers to implementing future-proofing strategies. This focus tends to prioritize quantity over quality, limiting the ability to plan for long-term adaptability. However, it is increasingly recognized that expanding bed numbers should not be seen as a marker of prestige or a reliable source of revenue. As the World Health Organization notes (2009), hospital beds are now viewed more as cost centers than assets, and should be complemented—or even replaced—by more meaningful indicators such as the range and quality of services provided. Short-term focus can lead to design decisions driven by immediate cost savings, which may obscure potential issues of overdesign and limit long-term adaptability (Jones et al., 2020). Effective future-proofing requires not only design strategies but also political will. Decision-makers should consider allowing budget flexibility, adopting a flexible brief, and shifting assurance procedures from capital costs to whole-life targets. Additionally, being aware of cognitive limits and personal commitments is crucial (Krystallis et al., 2019).

While some principles of future-proofing may be universally applicable, its implementation is inherently context-specific, necessitating further research to explore its implications and challenges across different countries. Additionally, as this study's findings are limited to architect interviews, future research is recommended to conduct case studies. While this study investigated the implementation and challenges of future-proofing based on architects’ perspectives—acknowledging their significant role as key actors—further research is needed to explore insights from other key stakeholders, such as clinicians, policymakers, and facility managers, to gain a more comprehensive understanding of the practical challenges and opportunities in future-proofing hospital buildings.

## Conclusion

This research, based on interviews with Australian hospital architects, reveals that future-proofing hospital buildings is a complex, context-sensitive endeavor shaped by a dynamic interplay of benefits, drawbacks, and implementation challenges. The findings reinforce that future-proofing is not a one-size-fits-all solution but a project-specific strategy that must navigate between long-term adaptability and immediate operational demands. While the concept of future-proofing is widely embraced, there remains limited evidence on its actual performance over time. The lack of systematic tracking of implemented strategies—particularly data on what has worked, what hasn’t, and under what conditions—continues to undermine confidence in its practical value. Despite many projects being labeled as future-proof, they often fall short in execution, underscoring the gap between aspiration and delivery.

To close this gap, a more structured, transparent, and evidence-informed approach is needed—one that empowers stakeholders, especially design teams, to evaluate, advocate for, and implement future-proofing strategies with greater clarity and accountability. This requires a shift from ad hoc, intuition-driven decision-making to a more deliberate framework grounded in strategic planning, foresight, systems thinking, and life cycle awareness. Importantly, future-proofing is not merely about anticipating change but about equipping hospitals to adapt over time without compromising core functions. It demands a delicate balance between preparing for multiple possible futures and managing pressing current realities. As this research highlights, understanding the full spectrum of benefits, trade-offs, and constraints can significantly enhance strategic decision-making in hospital design.

Given the persistent tendency to prioritize short-term political and economic pressures over long-term resilience, it is critical to adopt a more balanced planning paradigm—one that accommodates both present needs and future uncertainties. This study underscores the urgent need for longitudinal evaluations of future-proofing strategies, capturing their real-world performance over the life cycle of hospital buildings. Further research is also required to integrate advanced futures methodologies into design and planning processes, enabling more adaptive, context-aware, and strategically aligned healthcare infrastructure.

## Implications for Practice

- *Empower Architects to Inform Strategic Decision-Making:* Shift from focusing solely on initial capital costs to conducting comprehensive life cycle analyses, empowering architects to inform strategic decision-making by demonstrating the long-term benefits of future-proofing to stakeholders.
- *Integrate Futures Thinking Methodologies:* Incorporate future-thinking tools and techniques, such as scenario planning and back-casting, into the early planning and design phases.
- *Enhance Communication and Data Sharing:* Improve communication and data sharing across all project phases, including post-occupancy evaluations, to ensure that future-proofing strategies are understood and effectively implemented.
